# Common mitochondrial polymorphisms as risk factor for endometrial cancer

**DOI:** 10.1186/1755-7682-2-33

**Published:** 2009-10-28

**Authors:** Anna M Czarnecka, Aleksandra Klemba, Andrzej Semczuk, Katarzyna Plak, Barbara Marzec, Tomasz Krawczyk, Barbara Kofler, Pawel Golik, Ewa Bartnik

**Affiliations:** 1 Institute of Genetics and Biotechnology, Faculty of Biology, University of Warsaw, Pawinskiego 5a, 02-106, Warsaw, Poland; 2 School of Molecular Medicine, Medical University of Warsaw, Zwirki i Wigury 61, 02-091 Warsaw, Poland; 3 II Clinic and Ward of Gynecology, Medical University of Lublin, Lublin, Poland; 4 Department of Human Genetics, Lublin University School of Medicine, Lublin, Poland; 5 Clinical Pathology Laboratory, Monument Institute of Polish Mothers Health Center, Lodz, Poland; 6 Department of Pediatrics, University Hospital Salzburg, Paracelsus Medical University, Müllner Hauptstr 48, A-5020 Salzburg, Austria; 7 Institute of Biochemistry and Biophysics, Polish Academy of Sciences, Pawinskiego 5a, 02-106 Warsaw, Poland

## Abstract

Endometrial carcinoma is the most commonly diagnosed gynaecological cancer in developed countries. Although the molecular genetics of this disease has been in the focus of many research laboratories for the last 20 years, relevant prognostic and diagnostic markers are still missing. At the same time mitochondrial DNA mutations have been reported in many types of cancer during the last two decades. It is therefore very likely that the mitochondrial genotype is one of the cancer susceptibility factors. To investigate the presence of mtDNA somatic mutations and distribution of inherited polymorphisms in endometrial adenocarcinoma patients we analyzed the D-loop sequence of cancer samples and their corresponding normal tissues and moreover performed mitochondrial haplogroup analysis. We detected 2 somatic mutation and increased incidence of mtDNA polymorphisms, in particular 16223C (80% patients, p = 0.005), 16126C (23%, p = 0.025) and 207A (19%, p = 0.027). Subsequent statistical analysis revealed that endometrial carcinoma population haplogroup distribution differs from the Polish population and that haplogroup H (with its defining polymorphism - C7028T) is strongly underrepresented (*p *= 0.003), therefore might be a cancer-protective factor. Our report supports the notion that mtDNA polymorphisms establish a specific genetic background for endometrial adenocarcinoma development and that mtDNA analysis may result in the development of new molecular tool for cancer detection.

## Background

Endometrial cancer (EC) is the most frequently occurring invasive neoplasm of the female genital tract worldwide [1,2]. In 2007 approximately 39,000 new cases presented in the United States and 149,300 in Europe making it the fourth most common cancer among women. At the same time approximately 7400 of women were expected to die from this cancer in USA and 46 600 in Europe annually. These data enable to calculate that altogether 2.45% of women born today will be diagnosed with EC at some time during their lifetime [3,4].

Since 1988, the Gynecologic Oncology Committee of the International Federation of Gynecology and Obstetrics (FIGO) has recommended surgical staging of EC based on exploratory laparotomy, total abdominal hysterectomy, bilateral salpingo-oophorectomy, peritoneal cytology, and pelvic and para-aortic lymphadenectomy with the pathologic stage adding extra information. Unfortunately staging analysis still does not provide medical doctors, nor the patient with a relevant prognosis [5]. Multiple accessory prognostic factors have been defined for endometrial cancer, also including some molecular markers [2,6]. Nevertheless still the diagnosis is frequently uncertain because of false-positive rates of up to 25% and false-negative rates of up to 10% in cervical invasion evaluation with MRI *(*Magnetic Resonance Imaging) and sensitivity of invasion detection ranging from 66% to 100% (mean, 86%), and specificity from 92% to 100% (mean, 97%) [7]. Furthermore, the overall quality of surgical staging may be poor and very different from case to case as it is related to both the year that the surgeon passed the license examination and also to specialist status and experience [8]. It the face of presented data the need for new medical formation programmes and also novel diagnostic and prognostic markers is evident. Although endometrial carcinoma is associated with a good prognosis because patients tend to present with early disease, high-risk populations may benefit from screening, but no prospective studies have demonstrated a benefit in any population untill now. Therefore it is interesting to develop new screening tools that may enable to select populations at high EC risk and support the process of prevention and early diagnosis [9]. Until now *PTEN *(phosphatase and tensin homolog), K-*ras *(V-Ki-ras2 Kirsten rat sarcoma viral oncogene homolog), *TP53 *(tumor protein 53), *β-catenin*, *MSH2 *(MutS homolog 2, colon cancer, nonpolyposis type 2), *MSH6 *and Her2/neu (Human Epidermal growth factor Receptor 2); and mitochondrial gene mutations and protein signaling pathways have been implicated in the process of endometrial carcinogenesis [6,10,11]. On the basis of recent reports, it seems possible that a molecular mtDNA-analysis-based approach may be used in clinics in the future [6,12-19].

The first interest in mitochondrial function in carcinogenesis was reported as early as in the 1920s, when Otto Warburg discovered that cancer cells have a high glycolytic rate and produce increased levels of lactate in the presence of oxygen. Since then for more than two-thirds of the last century we have known that a common biochemical signature of many tumours, particularly those that are poorly differentiated and proliferate rapidly, is their propensity to utilize glucose at high rates [20,21]. This cancer characteristic has opened a new field of research today referred as to "mitochondrial medicine", since mitochondria are the metabolic organelles of the cells [22,23]. At this point the mitochondrial genome (mtDNA) came into the focus of multiple projects. mtDNA somatic mutations were described to arise in the cells of various types of human cancers including bladder, brain, breast, colon, head and neck, lung, ovarian, prostate, or thyroid [6,12,15,16,18,24-26]. At the same time inherited polymorphism have been pointed out as contributing factors in cancer development [24,27,28]. Nevertheless, the difficult task of correlating mtDNA polymorphisms and somatic mutations with neoplastic phenotype is not solved yet. The key role of the mitochondria in cell apoptotic pathways and the close link of tumour - suppressor proteins with mitochondria suggest some of the mechanisms of mitochondria dependent - tumourigenesis [6,18,19,25,26]. In particular, experiments on nude mice have shown that alterations in mtDNA (and a subsequent increase of ROS production) may contribute to cancer formation and development [16,29].

Our current research has been inspired by a number of studies indicating that mtDNA analysis may be more powerful in detecting tumour cells in bodily fluids and cytological specimens than nuclear DNA analysis [17,30]. Fliss and colleagues have reported facile analysis of mtDNA sequence in diagnostic samples [31]. In the present study, we examined the distribution of mtDNA inherited polymorphisms in the D-loop region of mtDNA in ECs population. Moreover we have also analyzed the presence of somatic mutations in primary ECs samples. In addition we have also investigated the distribution of mitochondrial haplogroups (haplogroup specific polymorphisms) in the patient population. Our choice of complementation of polymorphism analysis by haplogroup investigation was based on the rationale that the same mechanisms which may operate to create variation in evolution can also operate in clonal evolution in tumours [14]. Finally, in order to test for association with cancer susceptibility we compared mtDNA-data of EC patients with those for general Polish [32] and European populations [33,34].

## Results

### Haplogroup analysis

**Figure 1:**
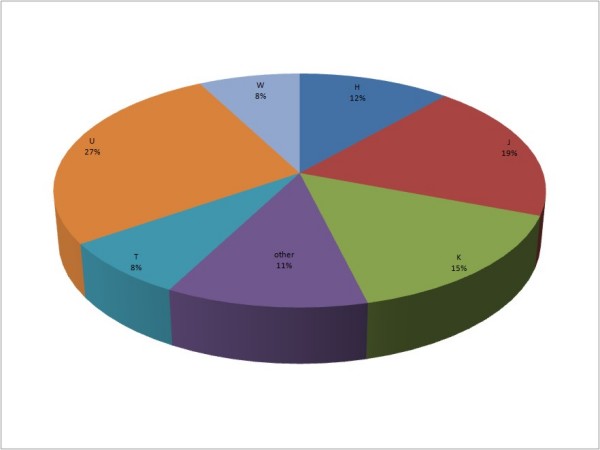
**Haplogroup distribution in the endometrial adenocarcinoma patient population**.

Altogether, our analysis on 26 patients has shown that seven (27%) belong to haplogroup U, five (19%) to haplogroup J, four (15%) to haplogroup K, three (12%) to haplogroup H, two (8%) to haplogroup T and two to haplogroup W (8%). No patient of haplogroup I, V or X was identified. In three (11%) cases, no haplogroup could be assessed due to unspecific polymorphisms found in haplogroup specific positions. Statistical analysis revealed that EC population haplogroup distribution is different from distribution found in the general Polish population. Most striking is the under-representation of haplogroup H individuals among EC patients (12 *vs*. 38%; *p *= 0.003). Other haplogroups seem to be represented in the cancer population at a frequency similar to the Polish population (Figure 1.).

As haplogroup H is characterized by T7028C polymorphism presence we have analyzed its statistical parameters. Patients with endometrial adenocarcinoma had the T7028C 12% of the time while in the general Polish population [32] carries the T7028C SNP 42% of the time. This difference is statistically significant p = 0.003 with Fisher's exact test, which was confirmed both by Yates corrected chi square χ^2 ^= 7.382 and p = 0.007 and 'N-1' Chi square χ^2 ^= 8.58 and p = 0.003, and these tests have been used as expected to give relatively low Type I error. The statistics was performed as previously suggested [35]. Moreover in cancer patients T7028C is found at RR = 0.28 (95% CI: 0.093<R.R.<0.812), which is 72% fewer than in non-cancer cohort. This gives OR = 0.18 and an inverse OR = 5.53, which means that one has a 5.53 higher chance to be T7028C carrier and not develop cancer in comparison to people who developed cancer, at specificity 0.775 (0.755 - 0.815). 23% more of 7028C do not develop endometrial adenocarcinoma in comparison to 7028T carriers. Negative Predictive Value (NPV) of 7028C test result is 0.581 (0.567 - 0.611) with Relative Risk Reduction (RRR) = 0.725 and NND = 5.69. 7028T carriers have RR = 4.51 of cancer development if compared with 7028C, with OR = 5.5316. The 7028T inheritance correlates with cancer development with Yules-Q of 0.694, and therefore indicates a moderately strong positive relationship. C7028T test has sensitivity of 0.885, which guarantees the recognition of the majority of cancer predisposition as such, thus a negative result may be used to rule out the disease susceptibility. High negative predictive value of this test at 0.950 (0.881 - 0.982 at 95% CI) also seems to confirms the utility of this test. At the same time a relatively low positive predictive value of 0.223 seems typical for a multi-factorial disease such as cancer. At the same time this test is cost-effective, NND is as low as 3.29. Inheritance of 7028T results in RRI of EC development as high as 350% in comparison to 7025C. At the same time ARI is 17.5%.

In face of those calculations 7028C is to have protective value in EC development with RRR = 78% and ARR = 18%. This percentage of 7028C corresponds to negative likelihood ratio of 0.225 (0.185 - 0.245 at 95% CI) and is considered to show a small decrease in the likelihood of disease. Diagnostic Odds Ratio of C7028T is 5.53 and as shown before DOR of 3 - 6 is hardly a useful test for diagnostic purposes, but it may provide a strong clue in investigations of etiology [36]. Nevertheless it has to be underlined that this research must be confirmed on large sample numbers to verify its relevance for clinical application. For evidence based medicine genotyping of a large number of patients is needed before any application can be suggested [17,37].

### Mutations in the D-loop mtDNA region

In the current study, the mtDNA D-loop sequence was analyzed by sequencing mtDNA from 26 cancerous and normal tissues. Both tissues were obtained from the patients with clinically diagnosed and pathologically confirmed EC during surgery. We were able to detect 2 somatic mutations (G16153A and A16188C). These genetic changes are point mutations and A16188C may be classified as mtMSI (mitochondrial satellite instability). As anticipated these mutations are located in mtDNA hypervariable region I (HV1 - 16024-16383). G16153A is a transition typically characteristic for mtDNA mutagenesis possibly induced by oxidative stress. In contrast, mtMSI might be generated during erroneous replication. These mutations seem to be relatively infrequent [12,15] and therefore possibly are more specific for the EC patient population.

### Germ-line polymorphisms in the D-loop mtDNA region

**Figure 2:**
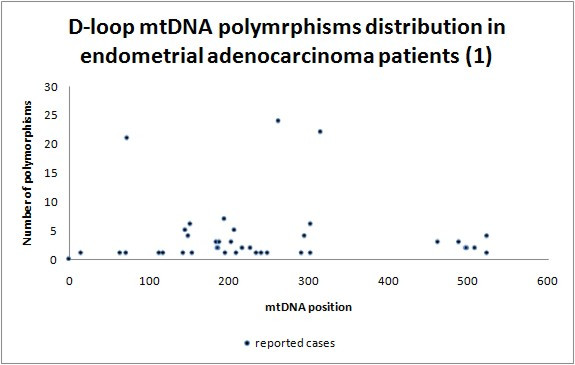
**D-loop nucleotides 1 - 576 polymorphism distribution in endometrial adenocarcinoma patients**.

**Figure 3:**
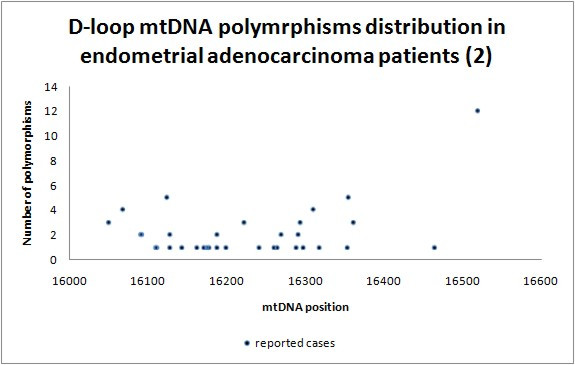
**D-loop 16024 - 16569 polymorphism distribution in endometrial adenocarcinoma patients**.

Our population of patients differs by 80 germ-line polymorphisms (Additional file 1; Table S1., Figure 2 and 3) from haplogroup H [38,39], the most common haplogroup in Poland, and the haplogroup we have just shown to be at lower risk for EC development [32,34]. Specifically only 22 out of 80 (28%) polymorphisms were generally common [33], and A16T polymorphism has not been reported previously [34]. We believe that polymorphisms uncommon in the global population, but abundant in the endometrial carcinoma cohort are good candidates for cancer-susceptibility loci. As predicted, polymorphisms were predominantly located in mtDNA hypervariable regions HV1 (16024-16383, Figure 3) and HV2 (57-333, Figure 2) - 34 (43%) and 27 (34%) polymorphisms, respectively.

Our special attention was aimed at 73, 143, 189, 199, 203, 204, 207, 250, 295, 16051, 16069, 16126, 16129, 16172, 16192, 16223, 16224, 16256, 16270, 16278, 16292, 16311, 16356, 16391 mtDNA position polymorphisms, as these are D-loop located haplogroup specific loci [40-42]. This analysis revealed an overabundance of specific polymorphisms in our cancer study population, in particular 16126C - specific for J or T haplogroups (p = 0.025); and 207A - typical for haplogroup W (p = 0.027). At the same time 16223T, specific for haplogroups W, X and I (p = 0.005) was underrepresented. In summary 16223C was present in 80% cases (21/26), 207A was found in 19% (5/26) and 16126C in 23% (6/26), so there might be a correlation and/or association between 16223C/207A genotype and EC development.

A number of patients harboured other unspecific polymorphisms, including 73A in patient 402 belonging to haplogroup T (G specific for haplogroup T), 16356C in patient 423 belonging to haplogroup U (T specific for haplogroup U), 16311C in patient 425 belonging to haplogroup W (C specific for haplogroups I, K, U, J and H) and 207A in patient 427 belonging to haplogroup K (G specific for haplogroup H) (data not shown). These polymorphisms would introduced a bias in haplogroup analysis if no data from RFLP and multiplex-PCR methods were available, which proves the need for whole-genome based analysis of haplogroup assignment.

### Polymorphisms in the coding region

In addition to D-loop polymorphism, our study reveals we were also able to detect 11 additional germ-line polymorphisms (Additional file 2; Table S2): four in 12S rRNA, one in ND1, ND2, ND3, CO3, tRNA-Arg, tRNA-Pro, which differ in our population from rCRS (haplogroup H). In this analysis polymorphism 4216C affirms the assignment of patients numbered 401, 403, 409, 410, 418, 428 to haplogroups J and T. Remarkably, position 10463, reported here as polymorphic, has been previously found subject to somatic mutation in EC by our group and A15960T has not been reported previously [43].

## Discussion

The use of mitochondrial DNA mutation and/or polymorphism patterns as a biomarker is rapidly expanding in disciplines ranging from rare metabolic diseases, aging, to cancer and the tracing of human migration patterns, population characterization, and human identification in forensic sciences [12,14,15]. In the case of endometrial adenocarcinoma the presence of mtDNA mutations has been analyzed by three independent research groups from China [44-46], and one from Italy [47]. All those studies have been rather limited. They included the analysis of: position 310 (6 cases) [47], nucleotide positions 514, 16189, and 16519 (51 cases) [48], D-loop region, the 12S and 16S rRNA genes (50 cases) [49], 12 microsatellite markers starting at nucleotide position 303, 514, 956, and 16184 (17 cases) [50], hypervariable segments of the control region, parts of 16S rRNA, tRNA(Leu) and ND1 gene (49 cases) [46]. In summary, all those papers reported one or more point mutations in 48.4% [50] to 56% of cases [40] and indicated mononucleotide repeat (D310) as a mutation 'hot-spot' in primary tumours [47]. In those reports no correlation between the pattern of mitochondrial abnormalities and clinical or histo-pathological features was found [47]. The mtDNA analysis of polymorphism distribution in endometrial adenocarcinoma was not as thorough and only the 16189T>C polymorphism was associated with EC susceptibility [48].

EC research [43,51] performed previously in our laboratory on a different patient cohort employed SSCP and eventually subsequent sequencing and covered ~10% of mtDNA, including nucleotides 135-433, 2986-3301, 4981-5500, 10390-10700 and 12005-12386. In this previously tested patient cohort, we found homoplasmic somatic mtDNA mutations in four out of 48 (8%) EC analyzed. These included alterations in ND2 (G5231A), ND4L (G10550A, T10640C, T10551C) and tRNAs (A12308G tRNA-Leu, C12258G tRNA-Ser, T10463C tRNA-Arg) [43,51]. Our current research on the D-loop reveals two mtDNA mutations (again only 8% patients) and shows multiple polymorphisms, including microsatellite instability in the C-tract (between nucleotides 303 and 315) in 22 (85%) of EC patients. The frequency of mtDNA mutations found in our previous analysis and in our new patient populations are similar, which strengthens the probability of low-mutation load in EC patients. Also in the report by Schwartz *et al*. mutations in the mitochondrial noncoding polycytidine (C)(n) repeat (polyC) analyzed in endometrium tumours (n = 53) were present in 11% of patients [52]. The frequency of D-loop mtDNA mutations reported by us and Schwartz *et al*. is much lower than that reported in other studies where the percentage was as high as 32.2% to 56% [49,50]. The frequency of mtDNA mutations in our study also differs from that reported in other human adenocarcinoma cases, including mutations found in 37% cases of gastric tumours [53], 40% - in Barrett's carcinomas [54], and 52% - in prostate cancers [55]. We believe that low number of mutations we detected is due to the stringent quality control we used [17,56] and reflects real number of mtDNA mutations in EC patients. In the light of that we believe that a great role in cell transformation must be attributed to inherited mtDNA polymorphisms.

A similar result of high polymorphism rate and low mutation load within cancer cells was reported in prostate [55] and thyroid cancer [57]. Also the risk of oral cancer was increased in patients bearing some of the common mitochondrial polymorphisms, not the mtDNA mutations [27]. As many germline mtDNA polymorphisms are found in the human population [12] it is therefore worthwhile to consider their differential occurrence in cancer-susceptible population with emphasis on their utility as potential cancer-predisposition markers [58,59]. In support of that hypothesis recent large review by the Wallace group revealed that a major part (72%) of previously reported tumour-specific somatic mtDNA mutations are actually mtDNA sequence variants found in the general population. The authors claim that 52% of the tumour somatic mRNA missense mutations, 83% of the tRNA, 38% of the rRNA, and 85% of the D-loop mutations are actually common sequence variants [12]. The report by Wallace and co-workers seems to favor our results of polymorphisms analysis.

Our sequencing analysis has been focused on the D-loop region of mtDNA. The mtDNA D-loop region is highly polymorphic, contains two hypervariable regions: HV1 (16024-16383) and HV2 (57-333) and was reported as somatic mutation "hot spot" in many types of cancer [60]. This directed our attention on this mtDNA region. We need to stress that D-loop contains crucial elements for mtDNA replication and transcription, and therefore sequence differences in this region might alter the rate of DNA replication by modifying the binding affinity of important trans-activating factors [61]. In fact, both down- and up-regulation of mtDNA replication was found in several tumour types [62,63]. Moreover it was shown that in some cases reduced copy number of mtDNA correlates with clinical outcome [64] and might be potentially used to predict prognosis [65]. In the above-mentioned papers the D-loop sequence variability was one of key contributing factors leading to decreased mtDNA level in breast tumours. In particular, the 303-315 stretch highly polymorphic in our patient cohort localizes in the conserved sequence block II (CBSII) and is the site of replication primer binding. Although it is not clear what the long-term impact of mtMSI in this locus is, it is known that replication efficacy is altered depending on the sequence length (poly-C polymorphisms), as a result of CSB II involvement in replication priming. CBSII heavy-strand contributes to formation of a persistent RNA-DNA hybrid that serves to prime mtDNA replication. The formation of RNA-DNA hybrids is dependent on the GC-rich element. Interestingly, efficient hybrid formation is also influenced by sequences 5' to the hybrid, including the CSBIII element [66]. Furthermore mtDNA transcription required to generate RNA primers used in the initiation of heavy strand DNA synthesis is critically dependent on the exact CSB II sequence [67]. Premature transcription termination occurs if particular mtMSI are found in positions 300 to 282 of the mtDNA sequence and may be completely abolished in the 319-289 mutants. In contrast, 304-300 mutants show a drastic decrease in transcription termination [68]. All these data support our hypothesis that mtDNA polymorphisms, in particular D-loop polymorphisms influence cell physiology and may result in a pro-carcinogenic phenotype of the carrier. In addition we believe that the physiological influence of D-loop (CA)(n) polymorphism is significant in a clinical perspective, not only in cell biology. This phenomenon was already identified in breast cancer population, where patients with multiple alleles of the mtDNA D-loop (CA)(n) polymorphism (state called heteroplasmy) had significantly poorer disease-free survival than those with one allele of the mtDNA D-loop (CA)(n) polymorphism. This result suggests that the mtDNA D-loop polymorphisms may be associated with cancer survival and we believe this might also be true for EC patients and should be investigated in nearest future [69].

The other position that was highly polymorphic in our research - 16189 has previously been in the focus of oncological research. The carriers of germ-line T to C polymorphism at 16189 are to be more susceptible to breast cancer and ganglioma development in the light of the high frequency of 16189C detected in cancer patients and low number of 16189C in healthy individuals [50]. Interestingly, in the report by Liu *et al*. the T16189C polymorphism was found in 14% of ECs. In addition this polymorphism was also linked with an increased risk of type II diabetes mellitus, which is well known to be a risk factor in the aetiology of endometrial and breast cancers [48,70]. In the screen of female cancers, deletions or insertions were detected in the poly C tract of tumour mtDNA and were linked in all cases to a germline T to C transition at 16189. The elimination of the T generates a long poly-C tract that is generally anticipated to exhibit higher instability. It was subsequently empirically indicated that this position represents a strong hotspot of mtMSI with a (C)_7-14 _pattern of variation [67]. Furthermore, nucleotides 16184 - 16193 are located on the 3'-end of a termination-associated sequence (TAS) and at the 7S DNA binding site which are thought to be involved in the regulation of mtDNA synthesis [71]. Again if polymorphisms at 16189 are in the focus, our hypothesis of mitochondrial polymorphism involvement in cancer development is again supported in many respects. The second part of our research focused on mtDNA mutation screen and we have detected A16188C which is also localized within 16184-16193 tract involved in the regulation of mtDNA synthesis. This locus was previously reported as mutated in breast cancer (16188 C→CC) [72] and listed as must-be-excluded in haplogroup analysis and usage of D-loop mutations as molecular clock [73].

The CCCCCTCCCC sequences located in the Hypervariable Regions I and II of the D-loop, but also the 12S rRNA gene (Additional file 2; Table S2.) emerge as instability 'hot spot' regions in endometrial carcinomas. Considering other mtDNA polymorphisms reported by us it is worth noting that some polymorphic sequences within mitochondrial tRNA genes (Additional file 2; Table S2.) may serve as unusual replication origins with pathogenic implications. Today we know that particular variants of tRNA genes may acquire secondary structures resembling mitochondrial origins of light strand replication, particularly structures that might invoke bi-directional replication. In consequence, this excessive replication may cause abundant mutations in genome regions not adapted to tolerate them [74]. At the same time context analysis for 303 polymorphisms revealed a complex influence of neighbouring bases on mutagenesis in the HVS I region. Analysis suggested that a transient misalignment dislocation mutagenesis operates in monotonous runs of nucleotides and plays an important role in generating base substitutions in mitochondrial DNA [75]. In our opinion all discussed mutation/polymorphism generation mechanisms seem to be in important in EC and have potential impact on cell physiology.

Finally we need to emphesize that as previously pointed by Salas and Bandelt [76] mtDNA sequencing and analysis techniques contain inherent problems, particularly with regard to the generation of authentic and useful data. In fact we understand that for the medical field it is important to employ standardized procedures based on scientific grounds, in order to have mtDNA-based evidence that may be accepted in clinics. Therefore we have put much effort in refinement of our amplification and sequencing strategies, as well as a posteriori quality control of mtDNA sequencing [17,77]. We believe that such sequencing efforts resulted in low number of mutations, as we have eliminated phantom mutations [78]. To further refine the authenticity of our sequencing results a posteriori, we utilized the strategy of focused database comparisons, as this method has been proven to be effective and successful in the case of modern mtDNA data [56,76,78]. Considering the critical analysis of mtDNA sequencing data [76,78] we would like to stress that our summary (additional file 2; Table S2 and additional file 3; Table S3.) should be treated and interpreted with caution, as probaly some previously reported cancer mutations we cite are actually misreported nucleotide variants [78,79].

The second part of our project covers haplogroup related polymorphism analysis (Additional file 4; Table S4.). The frequencies of mtDNA haplogroups vary between ethnic groups worldwide. Our strictly European population is exclusively distributed among the nine haplogroups designated: H, I, J, K, T, U, V, W, and X, whereas haplogroups A, B, C, D, and E that are characteristic for Asian population or haplogroups L1, L2, and L3 specific for the African population were not analyzed [42]. The research was inspired by the fact that some haplogroup specific polymorphisms are significantly correlated with increased or decreased risk of specific human disorders, for example - LHON and Parkinson's disease with haplogroup J [80,81], ALS with haplogroup I [82], AD in males with haplogroup U [83], and a significant increase in breast cancer development with haplogroup K [84]. Concurrently correlation between longevity and certain haplogroups (J and U) was reported [85] (Additional file 4; Table S4.). Those facts prompt us to think that haplogroup mtDNA variants could be specifically connected with cancer, in particular EC. We have chosen to thoroughly analyze haplogroups in our EC cohort, as polymorphism A5178C - characteristic for haplogroup D (typical for South-East Asia) was correlated with the development of EC. The woman carrying 5178C seems to be at a significantly higher risk of EC development [46]. Nevertheless the Chinese population has a different mitochondrial genetic background than the European or white American population, and as a result A5178C polymorphism - EC correlation is not exploitable or applicable as a clinical marker outside the region where it was observed. Until now there was no reported research on the haplogroup distribution in the Caucasian EC population (Medline^© ^search database). As we expected, the EC cohort haplogroup distribution was not similar to the healthy Polish population with underrepresented haplogroup H individuals among EC patients. We believe that this might suggest a protective role of polymorphisms typical for haplogroup H in cancer development. Our hypothesis seems plausible as mtDNA haplogroup H is very common in Caucasoids, reaching frequencies of ~50%, which certainly suggests that it may confer some advantage. Recent studies have suggested haplogroup H3 is highly protective against AIDS progression [13]. Moreover, mtDNA haplogroup H is a strong independent predictor of outcome during severe sepsis, conferring a over 2-fold increased chance of survival at 180 days compared with individuals with different haplogroups [86].

We believe that inherited mtDNA polymorphisms, both in the D-loop, and in the coding region (including haplo-group specific polymorphisms) may cause subtle differences in the encoded protein structure and function; and thus subtle changes in OXPHOS activity and free-radical production. It is therefore likely that mtDNA polymorphisms in mitochondrial genes involved in electron transport chain and oxidative phosphorylation result in increased oxidative stress and hypermutagenesis of mitochondrial as well as nuclear DNA. This predisposes an individual or population sharing the same mtDNA genotype to an earlier onset of degenerative cellular processes, such as the accumulation of somatic mtDNA variation, decline in OXPHOS capacity or faster cancer progression, as shown in a cybrid model [87].

## Conclusion

In conclusion we suggest that mitochondrial research will enable to establish bio-markers helping to identify individuals at high risk for developing specific cancer types and to develop screening approaches for early diagnosis of cancer [14,24,84,88]. Molecular assessment of mitochondrial abnormalities of cancer cells could represent a promising tool not only for prognosis and early diagnosis of neoplasia, but possibly also during the diagnosis or follow-up of cancer patients [15,16,19,89,90]. A number of studies have proven that the mitochondrial genome is more useful in detecting tumour cells in bodily fluids and cytological specimens than mutations in nuclear DNA [30] in bodily fluids [31] and we believe this can be applied to EC screening. Thus, we suggest that application of mtDNA polymorphism pattern analysis may be useful to select populations at increased risk of cancer development and in cancer patients render final diagnosis in those cases where the morphological abnormalities are unspecific or neoplastic cells are at difficult to detect. In those cases, the application of PCR-coupled with gel electrophoresis or DNA sequencing may lend itself to rapid analysis of multiple samples. In the clinical context, the high frequency of mitochondrial genome instability, in combination with PCR-based assays of high sensitivity, may be of potential clinical usefulness [22,56].

## Methods

### Patients

Altogether we investigated twenty-seven EC patients who had undergone surgery (total abdominal hysterectomy and bilateral oophorectomy) at the II^nd ^Department of Gynaecology, Medical University at Lublin, Lublin, Poland, between 2003 and 2006. Pelvic and para-aortic lymph node dissections were performed if material obtained at dilatation and curettage was diagnosed as non-endometrioid or poorly (G3) differentiated cancer, or when the neoplasm invaded over one-half of the myometrial thickness of the uterus at surgery. In selected cases, the patients underwent additional surgical procedures, including omentectomy, appendectomy, tumour cytoreduction or dissection of distant metastases. Non-neoplastic reference material was available in all cases. The patients received no chemotherapy, radiotherapy, or hormonal therapy before surgery. The clinical stage of the disease was classified according to the staging system of the FIGO [91]. The material was assessed histologically at the Department of Pathology, Medical University in Lublin, Lublin, Poland, based on the WHO classification [92]. The pathological findings were assessed with regard to histological type and grade, depth of myometrial infiltration, pattern of ovarian involvement, vascular space invasion, presence of the neoplastic material in the fallopian tube, and the presence of metastases in the ovaries and/or lymph nodes (Additional file 5; Table S5). summarizes the clinico-pathological features of EC patients enrolled in this research. The clinical records were reviewed in order to obtain information regarding patient age, menopausal status, presenting symptoms and surgical management.

Prior to the surgery, all patients had provided informed consent to the use of their post-surgical material. The project was approved by the local Ethics Committee at the Medical University of Warsaw, Warsaw, Poland (KB-0/6/2007 to AMC).

Control group (general Polish population) haplogroup distribution data has been derived from previous analyses of Polish population [32,93].

### Tissue collection and DNA isolation

At surgery, after the uterus was removed, the uterine corpus was gently cut and the neoplastic material was scraped to a sterile eppendorf tube. During this procedure, material collected from the uterine cavity was not cross-contaminated by cervical cells. Tissue obtained at surgery was sub-divided into two parts. One portion was fixed in buffered formalin (pH 7.4) for routine histopathological assessment, while the rest was immediately frozen in liquid nitrogen and stored at -80°C. Non-neoplastic material was also collected at surgery and stored at -80°C until assayed.

DNA was isolated by standard proteinase K treatment followed by phenol/chloroform/isoamyl alcohol extraction. DNA was precipitated with 0.3 M sodium acetate in 70% ethanol at -20°C overnight and resuspended in Tris-EDTA (TE) buffer (pH 8.0). DNA quantification was performed with measurements at an absorbance of 260 nm.

### Mutation and polymorphism analysis

A germline (inherited) polymorphism is described as a difference between normal tissue variant and the revised Cambridge Reference Sequence (rCRS) - present both in normal and tumor tissues. Whenever the change between sequences from tumor (investigated) sample and normal tissue occurs, it is defined as a somatic mtDNA mutation. Obtained alteration frequencies were compared with mtDB database data [33]. In all positive samples (mutation), sequencing reactions were validated by a new independent amplification and sequencing.

### Haplogroup analysis by RLFP

Common polymorphisms in mtDNA determining classes of related genotypes, referred to as haplogroups, were detected by restriction fragment length polymorphism (RFLP) analysis. Haplogroup RLFP analysis, restriction enzymes and primers used are summarized in Additional file 6; Table S6.

### Haplogroup analysis by multiplex-PCR/sequencing

To verify haplogroups established by RFLP (Additional file 7; Table S7 and Additional file 4; Table S4.), multiplex-PCR/sequencing was performed as described previously [94]. Moreover, haplogroups were also assigned based on specific D-loop polymorphisms according to published data [40-42]. If any unspecific RFLP or multiplex-PCR/sequencing variants were found, sequencing with appropriate primers listed in Additional file 8; Table S8. was performed in order to verify the mutation/polymorphism.

### PCR amplification of D-loop segment of mtDNA

mtDNA fragment (15587 - 964) containing the D-loop region (spanning nucleotide 16024 to 576) was amplified using eight pairs of primers. The primer pairs used and the sizes of the amplified products are shown in Additional file 7; Table S7.

Fifty-microlitre reactions contained 10 ng DNA and 0.5 μM primers, 0.2 mM each of deoxynucleotide triphosphate (dNTP), 1U of FIREPol^® ^DNA Polymerase (Solis BioDyne, Estonia) or *Pfu *DNA Polymerase (Fermentas AB, Lithuania) and 2.5 mM MgCl_2_. DNA was subjected to the following cycling conditions: initial denaturing at 95°C for 3 min followed by 94°C for 1 min, 55°C for 30 sec, and 72°C for 1 min for 40 cycles and final extension step at 72°C for 7 min. Two microlitres of PCR products was analysed on an ethidium bromide-stained, 3% agarose gel (40 min at 70 V) to demonstrate the presence of the amplification product and for its quantification.

### mtDNA sequence analysis

Sequence analysis was performed by: FinchTV Version 1.4.0 (Geospiza Inc., USA) and BioEdit version 7.0.5.3 (Copyright Tom Hall 1999-2007), contig assembly was performed with Sequencher 4.1.4 (Gene Codes Corporation, Ann Arbor, MI USA) and multiple sequence alignment was performed with Clustal W [95]. Normal and cancer tissue mtDNA sequences were compared with the revised Cambridge Reference Sequence (CRS) and sequence variants were recorded [38,39].

### Statistical analysis

Two tailed non-directional Fisher-Irwin (Fisher's exact test) was used for statistical analysis [96]. Statistical analysis was performed with PAST - PAlaeontological STatistics, *ver. 1.34 *(Øyvind Hammer, D.A.T. Harper and P.D. Ryan, 2005) and Analyse-it for Microsoft Excel General & Clinical Laboratory modules Version 1.73 (Analyse-it Software, Ltd. Copyright ^© ^1997-2005). The difference was considered statistically significant if *p *< 0.05. In selected cases, to confirm the result Fisher's test, Yates's chi and un-corrected chi squared test ('*N *- 1' chi squared test) have been used as expected to give relatively low Type I error in case of a small research cohort. The statistics was performed as previously suggested [35]. To further understand the significance of T7028C as a factor for favorable outcomes (odds ratio, relative risk, difference in proportions, absolute and relative reduction in risk) and of the effectiveness of a diagnostic criteria (number needed to diagnose, specificity, positive & negative predictive values, positive & negative likelihood ratios, diagnostic and error odds ratios) was performed. The parameters, as well as the confidence intervals for the estimated parameters are computed by a general method [97,98].

## Abbreviations

CSBII: conserved sequence block 2; EC: endometrial cancer; HVI: Hypervariable region I; mtDNA: mitochondrial DNA; ROS: Reactive Oxygen Species; RFLP: Restriction Fragment Length Polymorphism; mtMSI: mitochondrial satellite instability; RR: Relative Risk, Risk Ratio; OR: Odds Ratio; DOR: Diagnostic Odds Ratio; PPV: Positive Predictive Value; NPV: Negative Predictive Value; RRR: Relative Risk Reduction; ARR: Absolute Risk Reduction; RRI: Relative Risk Increase; ARI: Absolute Risk Increase; CI: Confidence Interval; NND: Number Needed to Diagnose.

## Competing interests

The authors declare that they have no competing interests.

## Authors' contributions

AMC, AS, BK, TK, PG, EB - have made substantial contributions to the conception and design of the research, AMC, EB, AS, BK, PG - have been involved in drafting the manuscript, AMC, AK, KP, BM - performed the research, AS - collected the patients. All authors read and approved the final manuscript.

## Supplementary Material

Additional file 1 **Table S1**. Germ-line polymorphisms in the D-loop region of mtDNA of the endometrial adenocarcinoma patients. Click here for file

Additional file 2 **Table S2**. Germ-line polymorphisms in the coding region of mtDNA of the endometrial adenocarcinoma patients. Click here for file

Additional file 3 **Table S3**. Statistical analysis of haplogroup distribution in endometrial carcinoma vs. general Polish population. Click here for file

Additional file 4 **Table S4**. Summary of mtDNA polymorphisms relevant to establish European haplogroups and its relation to clinical medicine. Click here for file

Additional file 5 **Table S5**. Clinical and pathological features of EC patients enrolled in the study. Click here for file

Additional file 6 **Table S6**. RLFP analysis data for haplogroup assignment; restriction enzymes and primers used in the study are indicated. Click here for file

Additional file 7 **Table S7**. Sequences of primers used for D-loop sequencing (listed according to start position in mtDNA). Click here for file

Additional file 8 **Table S8**. Sequences of the primers used for haplogroup analysis (listed according to start position in mtDNA). Click here for file
