# Supplementary material for: Common mitochondrial polymorphisms as risk factor for endometrial cancer
Source: Int Arch Med. 2009 Oct 28;2:33. doi: 10.1186/1755-7682-2-33 (PMC2775024; doi:10.1186/1755-7682-2-33)
Supplement: Additional file 1 — Table S1. Germ-line polymorphisms in the D-loop region of mtDNA of the endometrial adenocarcinoma patients. [file 1755-7682-2-33-S1.DOC]

**Table S6.** Germ-line polymorphisms in the D-loop region of mtDNA of the endometrial adenocarcinoma patients.

^a^ Unless indicated otherwise, the data are from MITOMAP [1] and mtDB [2] databases and the references therein.

| ***mtDNA position (CRS)*** | ***Polymorphism*** | ***Case No*** | ***No*** | ***A/G/C/T/del frequency (mtDB)*** | ***Tissues where sequence was found***  ***(MITOMAP)*** | ***P*** | ***Region/population where sequence variant predominantly found*** | ***References^a^*** |
| --- | --- | --- | --- | --- | --- | --- | --- | --- |
| **16** | **A>T** | 415 | 1 | 1865/0/0/**0** | **Nn** | **0.014** | **Nn** |  |
| **64** | C>T | 400 | 1 | 0/0/1838/**27** | aging brains | 0.343 | Africa, Japan, Autralia | [3] |
| **72** | T>C | 402 | 1 | 0/0/**71**/1794 | aging brains and POLG/PEO | 1.00 | Finland, Sweden, America, India, Sardinia | [3] |
| **73** | A>G | 400,401,  402,404,408,409, 410,411,412,413,415,417,418,420,421,422,423,425,426,427,428 | 21 | 309/**1555**/1/0 | aging brains, POLG/PEO & control muscle, *thyroid tumour, oral cancer* | 1.00 | Very common | [3, 4] |
| **114** | C>T | 413 | 1 | 0/0/1856/**9** | POLG/PEO muscle, oral cancer | 0.129 | *Extremely rare*, Ashkenazi Jew, India, haplotype E5H | [5, 6] |
| **119** | T>C | 425 | 1 | 0/0/**2**/1863 | Polymorphism | **0.0437** | *Extremely rare,* India |  |
| **143** | G>A | 420 | 1 | **25**/1840/0/0 | *oral cancer* | 0.322 | Asia, Autralia |  |
| **146** | T>C | 400,413,415,427,428 | 5 | 1/0/**190**/1674 | elderly fibroblasts, aging/AD brains, POLG/PEO & control muscle, *prostate tumour, ovarian carcinoma* | 0.369 | Africa, Japan, Taiwan, Finland, Italy, Spain, Algerian Jew, India, Polynesia, Caucasian | [7-10] |
| **150** | C>T | 404,408,411,415 | 4 | 0/2/**1616**/247 | elderly fibroblasts/leukocytes, *lung tumour, thyroid tumour* | 1.00 | China, Japan, Berbers, Italy |  |
| **152** | T>C | 400,407,409, 411,417,426 | 6 | 0/0/**396**/1469 | aging brains,elderly fibroblasts, *ovarian carcinoma, oral cancer* | 0.810 | Africa, China, Japan, American, Finland, Italy | [7, 10] |
| **155** | T>A | 410 | 1 | **0**/0/0/1865 | *oral cancer* | **0.014** | Nn |  |
| **185** | G>A | 410, 421,428 | 3 | **55**/1807/0/3 | POLG/PEO muscle, *thyroid tumour* | **0.0431** | Africa, China, Japan, American, Finland |  |
| **186** | C>A | 405,409 | 2 | **9**/0/1853/3 | Polymorphism | **0.011** | *Extremely rare,* Africa, Italy |  |
| **188** | A>G | 410,421 | 2 | 1845/**20**/0/0/ | *breast tumour* | **0.035** | *Extremely rare*, American, Finland |  |
| **189** | A>G | 420, 422, 425 | 3 | 1782/**75**/8/0 | elderly muscle, POLG/PEO muscle & fibroblasts, aging brains, *prostate tumour* | 0.088 | Japan, Finland, India | [7, 9] |
| **195** | T>C | 402,409, 411,412,420,422,423 | 7 | 11/0/**280**/1574 | elderly fibroblasts, *lung-cancer cells*, aging/AD brains, *thyroid tumour*, *oral cancer* | 0.338 | Africa, Japan, American, Finland, Italy, Caucasian | [4, 7, 8, 11] |
| **196** | T>C | 420 | 1 | 0/0/**4**/1861 | Polymorphism | 0.112 | *Extremely rare* , Japan,Finland,India |  |
| **204** | T>C | 422,425,420 | 3 | 0/0/**123**/1741 | oral cancer, *prostate tumour* | 0.446 | Japan, Finland, India | [9, 12] |
| **207** | G>A | 418,422,420,425,427 | 5 | **123**/1742/0/0 | *oral cancer, prostate tumour, thyroid tumour* | **0.027** | Japan, Finland, India | [4, 9] |
| **210** | A>G | 422 | 1 | 1854/**11**/0/0 | Polymorphism | 0.164 | *Extremely rare*, Japan |  |
| **217** | T>C | 417,426 | 2 | 0/0/**18**/1847 | Polymorphism | **0.029** | Japan, Finland, India |  |
| **228** | G>A | 410,421 | 2 | **57**/1805/0/3 | Polymorphism | 0.227 | American, Finland, India, Caucasian |  |
| **235** | A>G | 400 | 1 | 1805/**60**/0/0 | *prostate tumour* | 0.245 | Japan | [9] |
| **242** | C>T | 401 | 1 | 0/0/1854/**11** | POLG/PEO muscle | 0.603 | *Extremely rare*, American, Finland |  |
| **249** | DelA | 413 | 1 | Nn | Polymorphism | - | Nn |  |
| **263** | A>G | 400,401, 402,404,405,407,408, 409, 410,411,412,413,415,417,418,420,421,422,423,424,425,426,427,428 | 24 | 6/**1861**/0/0 | POLG/MNGIE muscle, oral cancer | 1.00 | Africa, Japan, China, Australia, American, Finland, India | [12] |
| **292** | T>C | 411 | 1 | 0/0/**0**/1867 | POLG/PEO muscle | **0.013** | Nn |  |
| **295** | C>T | 401,410,421,428 | 4 | 4/0/1788/**75** | POLG/MNGIE muscle | **0.021** | American, Finland, India, Caucasian |  |
| **303** | C7>C8  (ins) | 401,402,405, 410,422,426 | 6 | Nn | *multiple tumour types* | - | Africa, Japan, Taiwan, Finland, Italy, Spain, India, Polynesia, Caucasian, Ashkenazi Jew, American, Australia | [13] |
| **303** | C7TC5>C9TC6  (ins) | 411 | 1 | Nn | *multiple tumour types* | - | Africa, Japan, Taiwan, Finland, Italy, Spain, India, Polynesia, Caucasian, Ashkenazi Jew, American, Australia | [13] |
| **315** | C5>C6  (ins) | 400,401,402,404,407,408,409,410,411,412,413,415,417,418,420,421,422,424,425,426,427,428 | 22 | Nn | *multiple tumour types* | - | Africa, Japan, Taiwan, Finland, Italy, Spain, India, Polynesia, Caucasian, Ashkenazi Jew, American, Australia | [13] |
| **462** | C>T | 401,410,428 | 3 | 0/0/2073/**71** | *thyroid tumour* | 0.056 | American, Finland, India, Caucasian | [4] |
| **489** | T>C | 401,410,428 | 3 | 0/0/**777**/1367 | *ovarian carcinoma* | **0.022** | Africa, China, Japan, American, Finland, Italy, India | [10] |
| **497** | C>T | 413,415 | 2 | 0/0/2094/**49** | *thyroid tumour* | 0.147 | American, Ashkenazi Jew, Finland, Italy | [4, 14] |
| **499** | G>A | 412,423 | 2 | **38**/2106/0/0 | *thyroid tumour* | 0.081 | Japan | [4] |
| **508** | A>G | 417,426 | 2 | 2130/**14**/0/0 | Polymorphism | **0.014** | *Extremely rare,* American, Finland, Caucasian |  |
| **523** | AC5>AC4(del) | 400,402,407,411 | 4 | Nn | *ovarian carcinoma, thyroid tumours, gastric carcinomas* | - | Nn | [4, 10, 15] |
| **523** | AC5>AC6(ins) | 423 | 1 | Nn | *ovarian carcinoma & control tissue, thyroid tumours, breast tumours* | - | Nn |  |
| **16051** | A>G | 417,422,426 | 3 | 1829/**38**/0/0 | *oral cancer* | **0.017** | Africa, Japan, Finland, India |  |
| **16069** | C>T | 401,410, 421,428 | 4 | 0/0/1793/**73** | *oral cancer* | **0.019** | American, Caucasian, Finland, Italy |  |
| **16092** | T>C | 417,426 | 2 | 0/0/**22**/1845 | *oral cancer* | **0.041** | *Extremely rare*, Japan, India |  |
| **16093** | T>C | 402, 413 | 2 | 0/0/**98**/1768 | *oral cancer* | 0.644 | Japan, American, Finland, Italy, India |  |
| **16111** | C>T | 410 | 1 | 1/0/1830/**36** | colonic crypts somatic mutations, *oral cancer* | 0.426 | Japan | [16] |
| **16113** | A>C | 423 | 1 | 1867/0/**0**/0 | *oral cancer* | **0.013** | Nn |  |
| **16126** | T>C | 401,402,410,418,428 | 5 | 0/0/**166**/1701 | *oral cancer* | 0.202 | China, Japan, American, Finland, Italy, India |  |
| **16129** | G>C | 417,426 | 2 | 304/1554/**9**/0 | Polymorphism | **0.009** | Finland, Spain, India |  |
| **16129** | G>A | 415 | 1 | **304**/1554/9/0 | *oral cancer* | 0.106 | Arica, Japan, America, Italy |  |
| **16145** | G>A | 401 | 1 | **50**/1817/0/0 | *oral cancer* | 0.536 | Japan, American, Finland, Italy |  |
| **16163** | A>G | 409 | 1 | 1847/**20**/0/0 | Polymorphism | 0.269 | American, Finland, India |  |
| **16172** | T>C | 401 | 1 | 0/0/**150**/1717 | MNGIE tissues, head/neck tumour back-mutation, oral cancer | 0.721 | Japan, Morocco, Finland, Europe |  |
| **16176** | C>T | 409 | 1 | 2/2/1845/**18** | Polymorphism | 0.247 | Japan, Autralia |  |
| **16179** | C>T | 412 | 1 | 0/0/1862/**5** | Polymorphism | 0.079 | Asia, Austrlia |  |
| **16189** | C5TC4>C8TC | 404 | 1 | Nn | insulin resistance | - | Nn [17] |  |
| **16189** | C5TC4>C2TC7 | 409 | 1 | Nn | *prostate tumour* | - | Nn |  |
| **16189** | C5TC4>C10 | 411 | 1 | Nn | *endometrial tumour, familial breast caner* | - | Nn [18, 19] |  |
| **16189** | C5TC4>C11 | 417 | 2 | Nn | *endometrial tumour, familial breast caner* | - | Nn [18, 19] |  |
| **16201** | C>T | 422 | 1 | 0/0/1866/**1** | *oral cancer* | **0.027** | *Extremely rare*, Japan |  |
| **16223** | C>T | 400,422,425 | 3 | 0/0/992/**875** | *oral cancer* | **0.001** | Africa, Japan, China, Australia, India, Finland, Ashkenazi Jews |  |
| **16224** | T>C | 413, 415, 427 | 3 | 0/0/**107**/1760 | *oral cancer* | 0.254 | Japn, American, Finland, Ashkenazi Jews |  |
| **16242** | C>T | 400 | 1 | 1/0/1859/**7** | *oral cancer* | 0.104 | *Extremely rare,* Africa, Asia, Spain |  |
| **16261** | C>T | 401 | 1 | 0/0/1756/**111** | *oral cancer* | 1.00 | Japan, Taiwan, Finland, India |  |
| **16265** | A>G | 422 | 1 | 1842/**7**/16/2 | Polymorphism | 0.104 | *Extremely rare,* Japan, Finland, Italy |  |
| **16270** | C>T | 404,411 | 2 | 0/0/1802/**65** | *oral cancer* | 0.273 | Finland, Italy, India |  |
| **16290** | C>T | 400 | 1 | 0/0/1799/**68** | *breast tumour* | 1.00 | Japan |  |
| **16292** | C>T | 420,425 | 2 | 0/2/1801/**64** | *breast, ovarian, head/neck tumour, oral tumour* | 0.267 | Japan, Finland, Italy |  |
| **16294** | C>T | 409,418,422 | 3 | 0/0/1760/**107** | *oral cancer* | 0.189 | Japan, India, Finland, American |  |
| **16298** | T>C | 402 | 1 | 0/0/**169**/1698 | *oral cancer* | 0.724 | China, Japan, India, Finland, American |  |
| **16311** | T>C | 413,424,425,427 | 4 | 0/0/**340**/1526 | *oral cancer* | 0.637 | Africa, Japan, China, American, Finland, Italy, India |  |
| **16319** | G>A | 400 | 1 | **131**/1736/0/0 | *oral cancer* | 0.717 | China, Japan, India |  |
| **16355** | C>T | 400 | 1 | 5/0/1848/**14** | *oral cancer* | 0.187 | *Extremely rare*, Japan, Australian Aboriginee |  |
| **16356** | T>C | 407,411,412,417,423 | 5 | 0/0/**27**/1840 | *oral cancer* | **0.001** | India, Australian, Aboriginee |  |
| **16362** | T>C | 417,423,426 | 3 | 1/0/**444**/1422 | *oral cancer* | 0.168 | Africa, China Japan, Italy |  |
| **16465** | C>T | 411 | 1 | 0/0/1863/**4** | *ovarian tumour* | 0.066 | *Extremely rare*, Africa |  |
| **16519** | T>C | 408, 409, 410,412,415,413,418, 420, 421, 423  427,428 | 12 | 0/0/**1115**/752 | *oral cancer, gastric, lung, ovarian tumour* | 0.165 | Africa, Japan, Caucasian, China, American, Finland, Italy, India |  |

1. Brandon MC, Lott MT, Nguyen KC, Spolim S, Navathe SB, Baldi P, Wallace DC: **MITOMAP: a human mitochondrial genome database--2004 update**. *Nucleic Acids Res* 2005, **33**(Database issue):D611-613.

2. Ingman M, Gyllensten U: **mtDB: Human Mitochondrial Genome Database, a resource for population genetics and medical sciences**. *Nucleic Acids Res* 2006, **34**(Database issue):D749-751.

3. Jazin EE, Cavelier L, Eriksson I, Oreland L, Gyllensten U: **Human brain contains high levels of heteroplasmy in the noncoding regions of mitochondrial DNA**. *Proc Natl Acad Sci U S A* 1996, **93**(22):12382-12387.

4. Maximo V, Soares P, Lima J, Cameselle-Teijeiro J, Sobrinho-Simoes M: **Mitochondrial DNA somatic mutations (point mutations and large deletions) and mitochondrial DNA variants in human thyroid pathology: a study with emphasis on Hurthle cell tumors**. *Am J Pathol* 2002, **160**(5):1857-1865.

5. Del Bo R, Bordoni A, Sciacco M, Di Fonzo A, Galbiati S, Crimi M, Bresolin N, Comi GP: **Remarkable infidelity of polymerase gammaA associated with mutations in POLG1 exonuclease domain**. *Neurology* 2003, **61**(7):903-908.

6. Ruiz-Pesini E, Lott MT, Procaccio V, Poole JC, Brandon MC, Mishmar D, Yi C, Kreuziger J, Baldi P, Wallace DC: **An enhanced MITOMAP with a global mtDNA mutational phylogeny**. *Nucleic Acids Res* 2007, **35**(Database issue):D823-828.

7. Attardi G: **Role of mitochondrial DNA in human aging**. *Mitochondrion* 2002, **2**(1-2):27-37.

8. Coskun PE, Beal MF, Wallace DC: **Alzheimer's brains harbor somatic mtDNA control-region mutations that suppress mitochondrial transcription and replication**. *Proc Natl Acad Sci U S A* 2004, **101**(29):10726-10731.

9. Jeronimo C, Nomoto S, Caballero OL, Usadel H, Henrique R, Varzim G, Oliveira J, Lopes C, Fliss MS, Sidransky D: **Mitochondrial mutations in early stage prostate cancer and bodily fluids**. *Oncogene* 2001, **20**(37):5195-5198.

10. Liu VW, Shi HH, Cheung AN, Chiu PM, Leung TW, Nagley P, Wong LC, Ngan HY: **High incidence of somatic mitochondrial DNA mutations in human ovarian carcinomas**. *Cancer Res* 2001, **61**(16):5998-6001.

11. Fliss MS, Usadel H, Caballero OL, Wu L, Buta MR, Eleff SM, Jen J, Sidransky D: **Facile detection of mitochondrial DNA mutations in tumors and bodily fluids**. *Science* 2000, **287**(5460):2017-2019.

12. Tan DJ, Chang J, Chen WL, Agress LJ, Yeh KT, Wang B, Wong LJ: **Somatic mitochondrial DNA mutations in oral cancer of betel quid chewers**. *Ann N Y Acad Sci* 2004, **1011**:310-316.

13. Brandon M, Baldi P, Wallace DC: **Mitochondrial mutations in cancer**. *Oncogene* 2006, **25**(34):4647-4662.

14. Behar DM, Metspalu E, Kivisild T, Achilli A, Hadid Y, Tzur S, Pereira L, Amorim A, Quintana-Murci L, Majamaa K *et al*: **The matrilineal ancestry of Ashkenazi Jewry: portrait of a recent founder event**. *Am J Hum Genet* 2006, **78**(3):487-497.

15. Han CB, Li F, Zhao YJ, Ma JM, Wu DY, Zhang YK, Xin Y: **Variations of mitochondrial D-loop region plus downstream gene 1 2S rRNA-tRNA(phe) and gastric carcinomas**. *World J Gastroenterol* 2003, **9**(9):1925-1929.

16. Taylor RW, Barron MJ, Borthwick GM, Gospel A, Chinnery PF, Samuels DC, Taylor GA, Plusa SM, Needham SJ, Greaves LC *et al*: **Mitochondrial DNA mutations in human colonic crypt stem cells**. *J Clin Invest* 2003, **112**(9):1351-1360.

17. Gill-Randall R, Sherratt EJ, Thomas AW, Gagg JW, Lee A, Alcolado JC: **Analysis of a polycytosine tract and heteroplasmic length variation in the mitochondrial DNA D-loop of patients with diabetes, MELAS syndrome and race-matched controls**. *Diabet Med* 2001, **18**(5):413-416.

18. Wang Y, Liu VW, Ngan HY, Nagley P: **Frequent occurrence of mitochondrial microsatellite instability in the D-loop region of human cancers**. *Ann N Y Acad Sci* 2005, **1042**:123-129.

19. Wang Y, Liu VW, Tsang PC, Chiu PM, Cheung AN, Khoo US, Nagley P, Ngan HY: **Microsatellite instability in mitochondrial genome of common female cancers**. *Int J Gynecol Cancer* 2006, **16 Suppl 1**:259-266.
