# Supplementary material for: Common mitochondrial polymorphisms as risk factor for endometrial cancer
Source: Int Arch Med. 2009 Oct 28;2:33. doi: 10.1186/1755-7682-2-33 (PMC2775024; doi:10.1186/1755-7682-2-33)
Supplement: Additional file 2 — Table S2. Germ-line polymorphisms in the coding region of mtDNA of the endometrial adenocarcinoma patients. [file 1755-7682-2-33-S2.DOC]

**Table S7.** Germ-line polymorphisms in the coding region of mtDNA of the endometrial adenocarcinoma patients.

ND1 - NADH Dehydrogenase subunit 1, COIII - Cytochrome c oxidase subunit III, ND3 - NADH dehydrogenase subunit 3, ND4 - NADH dehydrogenase subunit 4, tRNA Pro - proline tRNA, tRNA Arg - arginine tRNA

| ***mtDNA position (CRS) and gene*** | ***Case No*** | ***CRS*** | ***Sequence found in patients*** | ***A/G/C/T***  ***frequency (mtDB)*** | ***P*** | ***Comments*** |
| --- | --- | --- | --- | --- | --- | --- |
| **663**  **12S rRNA** | 400 | A | G | 2618/**86**/0/0 | 0.570 | Found in Japan, native Americans, Italy |
| **709**  **12S rRNA** | 409, 418, 420, 425 | G | A | **444**/2260/0 | 1 | Found in Africa, native Americans, Japan, American, Finland, Italy, India |
| **750**  **12S rRNA** | 400, 401, 404, 405, 407, 408, 409, 410, 411, 412, 413, 415, 417, 418, 422, 423, 424, 425, 426, 427 | A | G | 22/**2682**/0/0 | 1 | CRS is a rare variant; G is very common in the whole world, A/G is consensus mutation |
| **1438**  **12S rRNA** | 416, 422 | A | G | 84/**2620**/0/0 | 1 | CRS is a rare variant; G is very common in the whole world, A/G is consensus mutation |
| **1719**  **16S rRNA** | 422 | G | A | **111**/2593/0/0 | 1 | G-A – *found in oral cancer* |
| **4216**  **ND1** | 401, 403, 409, 410, 418, 421,428 | T | C | 0/0/**244**/2460 | **0.009** | Found in native Americans, Finland, Italy, India Y→H, haplogroup J/T marker, *heteroplasmic in acute leukemia, homoplasmic or absent in earlier stage, sporadic parathyroid adenoma* |
| **9899**  **COIII** | 409 | T | C | 0/0/**28**/2676 | 0.243 | Synonymous change; found in native Americans, Finland, India; C very rare, T/C is synonymus mutation |
| **10143**  **ND3** | 409 | G | A | **10**/2694/0/0 | 0.100 | Extremely rare, changes G→S, found in native Americans, Finland, India. |
| **10463**  **tRNA Arg** | 409 | T | A | **0**/0/127/2577 | **0.009** | Found in *endometrial tumour* |
| **11953**  **ND4** | 401 | C | T | 0/0/2703/**1** | **0.018** | Patient variant extremely rare, L→L, T/C is synonymus mutation |
| **12007**  **ND4** | 401 | **G** | **A** | **96**/2608/0/0 | 0.611 | Tumour variant very common in the whole world; synonymous mutation; A found in *oral cancer* |
| **15960**  **tRNA Pro** | 407 | A | T | 2704/0/0/**0** | **0.009** | T not found in databases |
