# Supplementary material for: Common mitochondrial polymorphisms as risk factor for endometrial cancer
Source: Int Arch Med. 2009 Oct 28;2:33. doi: 10.1186/1755-7682-2-33 (PMC2775024; doi:10.1186/1755-7682-2-33)
Supplement: Additional file 3 — Table S3. Statistical analysis of haplogroup distribution in endometrial carcinoma vs. general Polish population. [file 1755-7682-2-33-S3.DOCX]

**Table S8.** Statistical analysis of haplogroups’ distribution in endometrial carcinoma vs. general Polish population.

|  | ***positive*** | ***negative*** | ***% positive*** | ***p vs Piechota [32]*** | ***p vs Malyarchuk [93]*** | ***p vs combined*** |
| --- | --- | --- | --- | --- | --- | --- |
| **H** | 3 | 23 | 0.12 | **0.012** | **0.001** | **0.001** |
| **I** | 0 | 26 | 0.00 | 1 | 1 | 1 |
| **J** | 5 | 21 | 0.19 | 0.080 | 0.058 | 0.049 |
| **K** | 4 | 22 | 0.15 | 0.101 | **0.017** | **0.026** |
| **other** | 3 | 23 | 0.12 | 0.094 | 0.054 | 0.054 |
| **T** | 2 | 24 | 0.08 | 0.742 | 0.755 | 0.757 |
| **U** | 7 | 19 | 0.27 | 0.608 | 0.172 | 0.198 |
| **V** | 0 | 26 | 0.00 | 0.371 | 0.622 | 0.389 |
| **W** | 2 | 24 | 0.08 | 0.155 | 0.269 | 0.222 |
| **X** | 0 | 26 | 0.00 | 1 | 1 | 1 |
| ***Total*** | **26** |  | **1** |  |  |  |
