# Supplementary material for: Common mitochondrial polymorphisms as risk factor for endometrial cancer
Source: Int Arch Med. 2009 Oct 28;2:33. doi: 10.1186/1755-7682-2-33 (PMC2775024; doi:10.1186/1755-7682-2-33)
Supplement: Additional file 4 — Table S4. Summary of mtDNA polymorphisms relevant to establish European haplogroups and its relation to clinical medicine. [file 1755-7682-2-33-S4.DOC]

**Table S5.** Summary of mtDNA polymorphism relevant to establish European hapologroups and its relation to clinical medicine.

| **Haplogroup** | **Restriction site** | **Enzyme** | **Polymorphism**  **(as to rCRS)** | **Other haplogroups where polymorphism is found (frequency in %, if known)** | **Disease involvement of haplogroup (associated with):** |
| --- | --- | --- | --- | --- | --- |
| **H** | -7025 | Alu I | (T)7028C [1-3] | -  (not C: I, J, K, M, T, U, V, W, X, A, B, C, D, E – 100%, H – 5.3%) [2] | increased activity of complex I, decrease the therapeutic response to riboflavin [4], increased lipoatrophy after HAART [5], increase Parkinson's disease risk [6], increase the penetrance of Alzheimer's disease [7], protective against ischemic stroke [8], promotes increased survival after sepsis [9], associated with dementia with Lewy bodies [10] |
|  | -14766 | Mse I | (T)14766C [2, 3] | B, I, K, A  (not C: K, M, T, U, V, W, X, A, C, D, E – 100%, H – 0.8%) [2, 11] |  |
| ***H1*** |  |  | G3010A [2] | D (100%), J (82%), L2, L3, U [2] |  |
| ***H2*** |  |  | 1438A [2] | I, L1, H (92%)  (not A: J, K, M, T, U, V, W, X, A, B, C, D, E – 100%) [2] |  |
|  |  |  | 4769A [2] | -  (not A: I, J, K, L, M, T, U, V, W, X, A, B, C, D, E – 100%, H – 93%) [2] |  |
| **I** | -1715 | Dde I | G1719A [1, 2] | X (100%), T, J, H [1, 2] | decrease risk of sporadic amyotrophic lateral sclerosis (ALS) [12], cluster JTWIX is associated with an increased risk of PD and the disease progression to dementia [13] |
|  | -4529 | Hae II | A4529T [1, 3] | H, I [14] |  |
|  | +8249 | Ava II | G8251A [1, 2] | W (100%), T [1, 2] |  |
|  | +10028 | Alu I | T10031C [1] | - |  |
|  | +10398 | Dde I | A10398G [1] | E, C, D, M and L (100%), J (87%), K (70%) [1, 2] |  |
|  | +16398 | Bam HI | G16398A [1] | - |  |
|  | +11961 | BsmF I | A11947G [3] | W [3] |  |
|  |  |  | T10238C [2] | - |  |
| **J** | -13704 | BstNI | G13708A [1-3] | A, B, H, L2, U, X (46%) [2] | accelerated progression of AIDS [15], and type 2 diabetes mellitus on-set [16], increase penetrance of LHON disease [17], and individual sensitivity to the ototoxic effect of cisplatin [18], cluster JTWIX is associated with an increased risk of PD and the disease progression to dementia [13], J predisposes to successful aging and longevity [19] |
|  | +10398 | Dde I | A10398G [1] | E, C, D, M and L (100%), I (93%), K (70%) [1, 2] |  |
|  | -16065 | Hinf I | C16069T [1] | - |  |
|  | +4220 | Nla III | T4216C [2] | T (100%), L2, H [2] |  |
|  |  |  | A12612G [2] | - |  |
| ***J1*** |  |  | G3010A [2] | D (100%), H (32%), L2, L3, U [2] |  |
| ***J2*** |  |  | C7476T [2] | - |  |
|  |  |  | G15257G [2] | K (6%) [2] |  |
| **K** | -9052 | Hae II | G9055A [1] | U | significant increase in the risk of developing breast cancer [20], familial amyloidosis with polyneuropathy early on-set [21], K1c increase Parkinson's disease (PD) risk [22], multiple sclerosis risk factor [23], cluster UKJT reduces the risk of PD [24] |
|  | +12308 | Hinf I | A12308G [1-3, 25] | U (100%) [1] |  |
|  | +10398 | Dde I | A10398G [1] | E, C, D, M and L (100%), I [1] (93%), J (87%), |  |
|  | -1806 | Psi I | A1811G [2] | U (36%), H [2] |  |
|  | +10497 | Nla III | A10550G [3] |  |  |
|  |  |  | G12372A [2] | U (100%), C [2] |  |
|  |  |  | G9055A [2] | U2, H [1, 2] |  |
|  |  |  | T14798C [26] |  |  |
| **T** | +13366 | Bam HI | G13368A [1, 2] | - | age-related macular degeneration (AMD) [27], coronary artery disease and diabetic retinopathy [28], cluster UKJT reduces the risk of PD [24], cluster JTWIX is associated with an increased risk of PD and the disease progression to dementia [13] |
|  | -4915 | Bfa I | A4917G [2, 3] | I [2] |  |
|  | +15606 | Alu I | A15607G [1, 2] | - |  |
|  | +15925 | MspI | G15928A [1, 2] | - |  |
|  | +4220 | Nla III | T4216C [2] | J (100%) [2] |  |
|  |  |  | G709A [2] | W (100%), L1 (54%), U, L3, K, H, B [2] |  |
|  |  |  | G1888A [2] | C (46%), A, J [2] |  |
|  |  |  | T10463C [2] | - |  |
|  |  |  | G14905A [2] | L3 [2] |  |
|  |  |  | G8697A |  |  |
| ***T1*** |  |  | C12633A [2] | - |  |
| ***T2*** |  |  | A11812G [2] | L1 [2] |  |
|  |  |  | A14233G [2] | I [2] |  |
| **U** | +12308 | Hinf I | A12308G [1-3] | K (100%) [1, 2] | accelerated progression of AIDS [15], significant decrease breast cancer risk [20], increase the penetrance of Alzheimer's disease [7], contribute to more severe progression of knee osteoarthritis [29], cluster UKJT reduces the risk of PD [24] |
|  |  |  | G12372A [2] | K (100%), C [2] |  |
| **U2** |  |  | A1811G [2] | K (98%), H [2] |  |
|  |  |  | G9055A [2] | K |  |
|  | +12308 | Hinf I | A12208G [2] | - |  |
|  |  |  | G12372A [2] | K (100%), C [2] |  |
| **U4** |  |  | A1811G [2] | K (98%), H [2] |  |
|  |  |  | T4646C [2] | H [2] |  |
|  |  |  | C11332T [2] | - |  |
| **U5** |  |  | T1397C [2] | - |  |
| **U5a** |  |  | A7768G [2] | - |  |
| **U5a1** |  |  | A14793G [2] | - |  |
| **U5b** |  |  | A5656G [2] | I, U [2] |  |
| **U6** |  |  | G7805A [2] | - |  |
|  |  |  | T14179C [2] | - |  |
| **V** | -4577 | Nla III | G4580A [1-3] | - |  |
|  | +15904 | Mse I | C15904T [2] | - |  |
|  | -14776 | Mse I | C14766T [3] | - |  |
| **W** | +8249 | Ava II | G8251A [1] | I (93%), T [1] | **increases penetrance of LHON disease [17], cluster JTWIX is associated with an increased risk of PD and the disease progression to dementia [13]** |
|  | -8994 | Hae III | G8994A [1] | - |  |
|  | +11961 | BsmF I | A11947G [3] | I [3] |  |
|  |  |  | G709A [2] | T (100%), L1 (54%), U, L3, K, H, B |  |
|  |  |  | T1243C [2] | - |  |
| **X** | -1715 | Dde I | G1719A [1] | I (100%), T, J, H [1, 2] | **cluster JTWIX is associated with an increased risk of PD and the disease progression to dementia [13]** |
|  | +6230 | Mnl I | T6221C [2] | L3 (55%), L1, T [2, 30] |  |
|  |  |  | T14470C [2] | A, B, H, U [2] |  |
| **M** | +10397 | Alu I | C10400T [1, 2] | C, D, E and M (100%) [2] | affects clinical expression of Leber’s hereditary optic neuropathy [31] |
|  |  |  | T10873C [2] | C, D, E, L1, L2, L3 and M (100%) [2] |  |

1. Torroni A, Huoponen K, Francalacci P, Petrozzi M, Morelli L, Scozzari R, Obinu D, Savontaus ML, Wallace DC: **Classification of European mtDNAs from an analysis of three European populations**. *Genetics* 1996, **144**(4):1835-1850.

2. Herrnstadt C, Elson JL, Fahy E, Preston G, Turnbull DM, Anderson C, Ghosh SS, Olefsky JM, Beal MF, Davis RE *et al*: **Reduced-median-network analysis of complete mitochondrial DNA coding-region sequences for the major African, Asian, and European haplogroups**. *Am J Hum Genet* 2002, **70**(5):1152-1171.

3. Ruiz-Pesini E, Lott MT, Procaccio V, Poole JC, Brandon MC, Mishmar D, Yi C, Kreuziger J, Baldi P, Wallace DC: **An enhanced MITOMAP with a global mtDNA mutational phylogeny**. *Nucleic Acids Res* 2007, **35**(Database issue):D823-828.

4. Di Lorenzo C, Pierelli F, Coppola G, Grieco GS, Rengo C, Ciccolella M, Magis D, Bolla M, Casali C, Santorelli FM *et al*: **Mitochondrial DNA haplogroups influence the therapeutic response to riboflavin in migraineurs**. *Neurology* 2009, **72**(18):1588-1594.

5. Hendrickson SL, Kingsley LA, Ruiz-Pesini E, Poole JC, Jacobson LP, Palella FJ, Bream JH, Wallace DC, O'Brien SJ: **Mitochondrial DNA haplogroups influence lipoatrophy after highly active antiretroviral therapy**. *J Acquir Immune Defic Syndr* 2009, **51**(2):111-116.

6. Khusnutdinova E, Gilyazova I, Ruiz-Pesini E, Derbeneva O, Khusainova R, Khidiyatova I, Magzhanov R, Wallace DC: **A mitochondrial etiology of neurodegenerative diseases: evidence from Parkinson's disease**. *Ann N Y Acad Sci* 2008, **1147**:1-20.

7. Fesahat F, Houshmand M, Panahi MS, Gharagozli K, Mirzajani F: **Do haplogroups H and U act to increase the penetrance of Alzheimer's disease?** *Cell Mol Neurobiol* 2007, **27**(3):329-334.

8. Rosa A, Fonseca BV, Krug T, Manso H, Gouveia L, Albergaria I, Gaspar G, Correia M, Viana-Baptista M, Simoes RM *et al*: **Mitochondrial haplogroup H1 is protective for ischemic stroke in Portuguese patients**. *BMC Med Genet* 2008, **9**:57.

9. Baudouin SV, Saunders D, Tiangyou W, Elson JL, Poynter J, Pyle A, Keers S, Turnbull DM, Howell N, Chinnery PF: **Mitochondrial DNA and survival after sepsis: a prospective study**. *Lancet* 2005, **366**(9503):2118-2121.

10. Chinnery PF, Taylor GA, Howell N, Andrews RM, Morris CM, Taylor RW, McKeith IG, Perry RH, Edwardson JA, Turnbull DM: **Mitochondrial DNA haplogroups and susceptibility to AD and dementia with Lewy bodies**. *Neurology* 2000, **55**(2):302-304.

11. Derbeneva OA, Starikovskaya EB, Wallace DC, Sukernik RI: **Traces of early Eurasians in the Mansi of northwest Siberia revealed by mitochondrial DNA analysis**. *Am J Hum Genet* 2002, **70**(4):1009-1014.

12. Mancuso M, Conforti FL, Rocchi A, Tessitore A, Muglia M, Tedeschi G, Panza D, Monsurro M, Sola P, Mandrioli J *et al*: **Could mitochondrial haplogroups play a role in sporadic amyotrophic lateral sclerosis?** *Neurosci Lett* 2004, **371**(2-3):158-162.

13. Autere J, Moilanen JS, Finnila S, Soininen H, Mannermaa A, Hartikainen P, Hallikainen M, Majamaa K: **Mitochondrial DNA polymorphisms as risk factors for Parkinson's disease and Parkinson's disease dementia**. *Hum Genet* 2004, **115**(1):29-35.

14. Maca-Meyer N, Gonzalez AM, Larruga JM, Flores C, Cabrera VM: **Major genomic mitochondrial lineages delineate early human expansions**. *BMC Genet* 2001, **2**:13.

15. Hendrickson SL, Hutcheson HB, Ruiz-Pesini E, Poole JC, Lautenberger J, Sezgin E, Kingsley L, Goedert JJ, Vlahov D, Donfield S *et al*: **Mitochondrial DNA haplogroups influence AIDS progression**. *AIDS* 2008, **22**(18):2429-2439.

16. Feder J, Ovadia O, Blech I, Cohen J, Wainstein J, Harman-Boehm I, Glaser B, Mishmar D: **Parental diabetes status reveals association of mitochondrial DNA haplogroup J1 with type 2 diabetes**. *BMC Med Genet* 2009, **10**:60.

17. Shafa Shariat Panahi M, Houshmand M, Tabassi AR: **Mitochondrial D-loop variation in leber hereditary neuropathy patients harboring primary G11778A, G3460A, T14484C mutations: J and W haplogroups as high-risk factors**. *Arch Med Res* 2006, **37**(8):1028-1033.

18. Peters U, Preisler-Adams S, Lanvers-Kaminsky C, Jurgens H, Lamprecht-Dinnesen A: **Sequence variations of mitochondrial DNA and individual sensitivity to the ototoxic effect of cisplatin**. *Anticancer Res* 2003, **23**(2B):1249-1255.

19. Rose G, Passarino G, Carrieri G, Altomare K, Greco V, Bertolini S, Bonafe M, Franceschi C, De Benedictis G: **Paradoxes in longevity: sequence analysis of mtDNA haplogroup J in centenarians**. *Eur J Hum Genet* 2001, **9**(9):701-707.

20. Bai RK, Leal SM, Covarrubias D, Liu A, Wong LJ: **Mitochondrial genetic background modifies breast cancer risk**. *Cancer Res* 2007, **67**(10):4687-4694.

21. Olsson M, Hellman U, Plante-Bordeneuve V, Jonasson J, Lang K, Suhr OB: **Mitochondrial haplogroup is associated with the phenotype of familial amyloidosis with polyneuropathy in Swedish and French patients**. *Clin Genet* 2009, **75**(2):163-168.

22. Gaweda-Walerych K, Maruszak A, Safranow K, Bialecka M, Klodowska-Duda G, Czyzewski K, Slawek J, Rudzinska M, Styczynska M, Opala G *et al*: **Mitochondrial DNA haplogroups and subhaplogroups are associated with Parkinson's disease risk in a Polish PD cohort**. *J Neural Transm* 2008, **115**(11):1521-1526.

23. Hassani-Kumleh H, Houshmand M, Panahi MS, Riazi GH, Sanati MH, Gharagozli K, Ghabaee M: **Mitochondrial D-loop variation in Persian multiple sclerosis patients: K and A haplogroups as a risk factor!!** *Cell Mol Neurobiol* 2006, **26**(2):119-125.

24. Pyle A, Foltynie T, Tiangyou W, Lambert C, Keers SM, Allcock LM, Davison J, Lewis SJ, Perry RH, Barker R *et al*: **Mitochondrial DNA haplogroup cluster UKJT reduces the risk of PD**. *Ann Neurol* 2005, **57**(4):564-567.

25. Fraumene C, Belle EM, Castri L, Sanna S, Mancosu G, Cosso M, Marras F, Barbujani G, Pirastu M, Angius A: **High resolution analysis and phylogenetic network construction using complete mtDNA sequences in sardinian genetic isolates**. *Mol Biol Evol* 2006, **23**(11):2101-2111.

26. Brandstatter A, Parsons TJ, Parson W: **Rapid screening of mtDNA coding region SNPs for the identification of west European Caucasian haplogroups**. *Int J Legal Med* 2003, **117**(5):291-298.

27. SanGiovanni JP, Arking DE, Iyengar SK, Elashoff M, Clemons TE, Reed GF, Henning AK, Sivakumaran TA, Xu X, DeWan A *et al*: **Mitochondrial DNA variants of respiratory complex I that uniquely characterize haplogroup T2 are associated with increased risk of age-related macular degeneration**. *PLoS One* 2009, **4**(5):e5508.

28. Kofler B, Mueller EE, Eder W, Stanger O, Maier R, Weger M, Haas A, Winker R, Schmut O, Paulweber B *et al*: **Mitochondrial DNA haplogroup T is associated with coronary artery disease and diabetic retinopathy: a case control study**. *BMC Med Genet* 2009, **10**:35.

29. Rego-Perez I, Fernandez-Moreno M, Fernandez-Lopez C, Arenas J, Blanco FJ: **Mitochondrial DNA haplogroups: role in the prevalence and severity of knee osteoarthritis**. *Arthritis Rheum* 2008, **58**(8):2387-2396.

30. Reidla M, Kivisild T, Metspalu E, Kaldma K, Tambets K, Tolk HV, Parik J, Loogvali EL, Derenko M, Malyarchuk B *et al*: **Origin and diffusion of mtDNA haplogroup X**. *Am J Hum Genet* 2003, **73**(5):1178-1190.

31. Ji Y, Zhang AM, Jia X, Zhang YP, Xiao X, Li S, Guo X, Bandelt HJ, Zhang Q, Yao YG: **Mitochondrial DNA haplogroups M7b1'2 and M8a affect clinical expression of leber hereditary optic neuropathy in Chinese families with the m.11778G-->a mutation**. *Am J Hum Genet* 2008, **83**(6):760-768.
