# Supplementary material for: Common mitochondrial polymorphisms as risk factor for endometrial cancer
Source: Int Arch Med. 2009 Oct 28;2:33. doi: 10.1186/1755-7682-2-33 (PMC2775024; doi:10.1186/1755-7682-2-33)
Supplement: Additional file 5 — Table S5. Clinical and pathological features of EC patients enrolled in the study. [file 1755-7682-2-33-S5.DOC]

## Table S1. Clinical and pathological features of EC patients enrolled in the study.

| **Parameter** | **Number of cases**  **n (%)** |
| --- | --- |
| **Patient age (years)**  **<50**  **>50** | 4(15)  22(85) |
| **FIGO stage**  **I**  **II**  **III**  **IV** | 18(70)  4(15)  3(11)  1(4) |
| **Histologic type**  **endometrioid EC**  **non-endometrioid EC** | 25(96)  1(4) |
| **Histologic grade**  **G1**  **G2**  **G3** | 11(41)  7(29.5)  8(29.5) |
| **Myometrial invasion**  **none**  **<1/2**  **>1/2** | 2(8)  9(33)  15(59) |
| **VSI**  **positive**  **negative** | 9(33)  17(67) |
| **Coexistence of hyperplastic and neoplastic endometrium**  **positive**  **negative** | 6(22)  20(78) |
| **Presence of the neoplasm in the**  **fallopian tube**  **positive**  **negative** | 2(8)  24(92) |
| **Presence of the neoplasm**  **in the uterine cervix**  **positive**  **negative** | 7(25)  19(75) |
| **Metastases to the ovary/ovaries**  **positive**  **negative** | 2(8)  24(92) |
| **Lymph node metastasis***  **positive**  **negative** | 2(10)  17(90) |
| **Total** | **26(100)** |
