# Supplementary material for: Common mitochondrial polymorphisms as risk factor for endometrial cancer
Source: Int Arch Med. 2009 Oct 28;2:33. doi: 10.1186/1755-7682-2-33 (PMC2775024; doi:10.1186/1755-7682-2-33)
Supplement: Additional file 6 — Table S6. RLFP analysis data for haplogroup assignment; restriction enzymes and primers used in the study are indicated. [file 1755-7682-2-33-S6.DOC]

## Table S2. RLFP analysis data for haplogroups assignment with restriction enzymes and primers in the study used indicated.

| **Haplogroup** | **Enzyme** | **mtDNA sequence (CRS)/primers** | **Polymorphism of interest (CRS)** | **Other restriction sites in amplified sequence (mtDNA)** |
| --- | --- | --- | --- | --- |
| **H** | AluI | 6730F – 7398R | **7025** (restriction site lost) | 6867, 7055 |
| **U** | HinfI | 11902F –12328R | **12308** | 12123,12170 |
| **K** | HinfI | 11902F –12328R | **12308** | 12123,12170 |
| **K** | HaeII | 8563F – 9231R | **9052** (restriction site lost) | 9052 |
| **I** | BamHI | 15879F –16545R | **16389** | - |
| **I** | AluI | 9821F – 10516R | **10028** | 10232 |
| **I** | AvaII | 7960F – 8641R | **8249** | - |
| **I** | HaeII | 4184F – 4869R | **4529** (restriction site lost) | 4533 |
| **W** | HaeIII | 8563F – 9231R | **8994** (restriction site lost) | 8573, 8839, 8895, 9026 |
| **T** | Alu I | 15372F – 16067R | **15606** | 15776 |
| **T** | BamHI | 12951F – 13614R | **13366** | - |
| **J** | BstnI | 13568F – 14276R | **13704** (restriction site lost) | 13704 |
| **J** | HinfI | 15879F – 16545R | **16065** (restriction site lost) | 16065 |
| **V** | NlaIII | 4184F – 4869R | **4577** (restriction site lost) | 4864 |
| **X** | DdeI | 1138F – 1801R | **1715** (restriction site lost) | 1248, 1436, 1450, 1637, 1667 |
