# Supplementary material for: Common mitochondrial polymorphisms as risk factor for endometrial cancer
Source: Int Arch Med. 2009 Oct 28;2:33. doi: 10.1186/1755-7682-2-33 (PMC2775024; doi:10.1186/1755-7682-2-33)
Supplement: Additional file 7 — Table S7. Sequences of primers used for D-loop sequencing (listed according to start position in mtDNA). [file 1755-7682-2-33-S7.DOC]

**Table S4.** Sequences of primers used for D-loop sequencing (listed according to start position in mtDNA).

| **Primer** | **primer sequence (5’ – 3’)** | **amplified mtDNA region (CRS)** |
| --- | --- | --- |
| **108F** | AGCACCCTATGTCGCAGTATC | 108 – 638 |
| **276R** | TCTGTGTGGAAAGTGGCTGTG | 16344 – 276 |
| **315F** | CGCTTCTGGCCACAGCAC | 315 – 803 |
| **548F** | CCAACCAAACCCCAAAGAC | 548 – 964 |
| **559R** | GGGTTTGGTTGGTCCGGG | 16495 – 559 |
| **638R** | GGTGATGTGAGCCCGTCTAAAC | 108 – 638 |
| **803R** | GGTGTGGCTAGGCTAAGC | 315 – 803 |
| **964R** | GGGAGGGGGTGATCTAAAAC | 548 – 964 |
| **15587F** | CTCCGATCCGTCCCTAACAAAC | 15587 – 16185 |
| **15879F** | AATGGGCCTGTCCTTGTAG | 15879 – 16545 |
| **16098F** | ACATTACTGCCAGCCACCATG | 16098 – 16456 |
| **16185R** | GGTTTTGATGTGGATTGGGT | 15587 – 16185 |
| **16344F** | CAGTCAAATCCCTTCTCGTCCC | 16344 – 276 |
| **16456R** | CCGGAGCGAGGAGAGTAGC | 16098 – 16456 |
| **16495F** | CGACATCTGGTTCCTACTTC | 16495 – 559 |
| **16545R** | AACGTGTGGGCTATTTAGGC | 15879 – 16545 |
