# Supplementary material for: Common mitochondrial polymorphisms as risk factor for endometrial cancer
Source: Int Arch Med. 2009 Oct 28;2:33. doi: 10.1186/1755-7682-2-33 (PMC2775024; doi:10.1186/1755-7682-2-33)
Supplement: Additional file 8 — Table S8. Sequences of the primers used for haplogroup analysis (listed according to start position in mtDNA). [file 1755-7682-2-33-S8.DOC]

**Table S3.** Sequences of the primers used for haplogroup analysis (listed according to start position in mtDNA).

| **Primer** | **primer sequence (5’ – 3’)** | **amplified mtDNA region (CRS)** |
| --- | --- | --- |
| **1138F** | GAACACTACGAGCCACAGC | 1138 – 1156 |
| **1801R** | TCATCTTTCCCTTGCGGTAC | 1801 – 1782 |
| **4184F** | TCCTACCACTCACCCTAGC | 4184 – 4202 |
| **4869R** | GTCATGTGAGAAGAAGCA | 4869 – 4852 |
| **6730F** | CTATGATATCAATTGGCTTCC | 6730 – 6750 |
| **7398R** | GGCATCCATATAGTCACTCC | 7398 – 7379 |
| **7960 F** | ATTATTCCTAGAACCAGGCG | 7960 – 7979 |
| **8563F** | ACAATCCTAGGCCTACCCG | 8563 – 8581 |
| **8641R** | TGATGAGATATTTGGAGGTGG | 8641 – 8621 |
| **9231R** | GATAGGCATGTGATTGGTGG | 9231 – 9212 |
| **9821F** | ACTTCACGTCATTATTGGCTC | 9821 – 9841 |
| **10516R** | ATGGAGATGGTAATTGCTAG | 10516 – 10497 |
| **11902F** | GCTAGTCCACGTTCTCCT | 11902 – 11921 |
| **12328R** | TTTGGAGTTGCACCAAGAATT | 12328 – 12309 |
| **12951F** | CGCTAATCCAAGCCTCACC | 12951 – 12969 |
| **13568F** | TTACTCTCATCGCTACCTCC | 13568 – 13587 |
| **13614R** | TATTCGAGTGCTATAGGCGC | 13614 –13596 |
| **14276R** | GGTTGATTCGGGAGGATCC | 14276 – 14258 |
| **15372F** | TAGGAATCACCTCCCATTCC | 15372 – 15391 |
| **15879F** | AATGGGCCTGTCCTTGTAG | 15879 – 15897 |
| **16067R** | GTCAATACTTGGGTGGTACC | 16067 – 16048 |
| **16545R** | AACGTGTGGGCTATTTAGGC | 16545 – 16526 |
